# Immunologic Biomarkers for Clinical and Therapeutic Management of Psoriasis

**DOI:** 10.1155/2014/236060

**Published:** 2014-07-20

**Authors:** P. Cordiali-Fei, L. Bianchi, C. Bonifati, E. Trento, M. Ruzzetti, F. Francesconi, S. Bultrini, G. D'Agosto, V. Bordignon, V. Francavilla, A. Tripiciano, A. Chiricozzi, E. Campione, C. Cavallotti, A. Orlandi, E. Berardesca, A. Di Carlo, S. Chimenti, F. Ensoli

**Affiliations:** ^1^Clinical Pathology & Microbiology, San Gallicano Dermatology Institute, Via Elio Chianesi 53, 00144 Rome, Italy; ^2^Dermatology, Tor Vergata University, Via Montpellier 1, 00133 Rome, Italy; ^3^Clinical Dermatology, San Gallicano Dermatology Institute, Via Elio Chianesi 53, 00144 Rome, Italy; ^4^Anatomic Pathology, Tor Vergata University, Via Montpellier 1, 00133 Rome, Italy

## Abstract

*Background*. The therapeutic management of psoriasis includes conventional treatments as well as the new generation of highly effective TNF-*α* inhibitors. However, psoriasis has proven to be a complex therapeutic challenge and treatment failures are not uncommon. Thus, laboratory biomarkers of disease progression/therapeutic efficacy may greatly help in the clinical management of psoriasis. *Aims*. To identify laboratory biomarkers for clinical management and therapeutic monitoring of psoriasis. *Methods*. An observational study performed on 59 patients, presenting moderate to severe psoriasis, undergoing treatment with anti-TNF-*α* agents (etanercept, adalimumab, and infliximab). Soluble and cellular immune/inflammatory parameters were assessed at baseline and after 12 and 24 weeks of treatment. *Results*. Clinical efficacy was achieved in 88% of the subjects at 12 weeks, reaching 90% after 24 weeks. IL-6 and IL-22, which were elevated at baseline, were significantly reduced, in association with a significant decrease of CLA+ T cells and an increase of Treg lymphocytes. T, B, and NK cell subsets and T cell response to recall antigens did not show any evidence of immune suppression. *Conclusions*. Immune/inflammatory parameters including IL-6 and IL-22, CLA+ T cells, and Treg lymphocytes may prove to be valuable laboratory tools for the clinical and therapeutic monitoring of psoriasis.

## 1. Introduction 

Psoriasis is an inflammatory and hyperproliferative disease of the skin and joints of unknown etiology, however characterized by a multifactorial genetic basis [1, 2]. Psoriasis has a strong genetic component; there is a positive family history in a high percentage of affected children [3, 4] and candidate genes linked to psoriasis are associated with the acquired or innate arms of the immune system [5–7]. Psoriasis is considered an autoimmune disorder mediated by T cells which, after priming by bacterial antigens, migrate to the skin where they are activated by self-antigens expressed by epithelia [5, 8] and the key participation of dendritic cells that appear also increased in the skin [9]. Soluble biologic mediators produced by activated T cells or by dendritic cells, such as interferon *γ* (IFN-*γ*), tumor necrosis factor-*α* (TNF-*α*), IL-17, and IL-22, can induce inflammatory molecules and angiogenic factors in keratinocyte and alter their differentiation pathway [5, 10]. These events lead to the cutaneous expression of the disease which consists in large erythematous desquamative plaques. However, psoriasis is frequently associated with the development of arthritis (PsA), due to focal bone erosion and repair processes consequent to inflammatory involvement of peripheral or axial joints [11, 12]. A key role in development of psoriatic lesions appears to be due to the expression of homing receptors by activated T cells, facilitating their migration to the skin [13]. Among them, the cutaneous lymphocyte-associated antigen (CLA), an epitope generated by fucosyltransferase-VII mediated modification of P-selectin glycoprotein ligand-1, which binds to E-selectin on vascular endothelium in the skin [14], might be particularly relevant for the pathogenesis of the disease, since evolving psoriatic skin lesions have been described to be enriched with CLA+ T cells when compared with stable lesions and distant uninvolved skin [15]. A strong positive correlation has been found between the frequency of CLA+ T cells in the blood of untreated psoriasis patients and their disease severity [16]. As a result, a complex cytokine network appears activated in psoriatic lesions. This includes elevated levels of IFN-*γ*, TNF-*α*, several interleukins (IL-1, IL-2, IL-6, IL-8, IL-12 (p40 subunit), IL-17, and IL-19), and multiple chemokines [6, 7]. Among the inflammatory molecules influencing the keratinocytes, TNF-*α* appears critical in sustaining most of the clinical manifestations of psoriasis, at both the joint and skin localizations. TNF-*α* is secreted by effector cells belonging to either the innate or the adaptive immune system and is capable of amplifying the response of both systems with pleiotropic modalities [17]. This notion is reinforced by the results of clinical trials based on the administration of anti-TNF-*α* therapy in patients with psoriatic arthritis [18–21]. A further link between altered immunologic circuitries, lymphocytes infiltration, and epidermal hyperplasia has been provided by recent studies which show that T cells expressing IL-17 may play a major role in psoriasis [22, 23]. This pathological immune circuitry appears driven by interleukin-23 [24]. In mice, injection of IL-23 leads to epidermal hyperplasia mediated by IL-22 which, in turn, is produced by IL-17-expressing T cells [25]. A similar scenario is suggested by study in humans [26, 27]. On the other hand, an impairment of regulatory T lymphocytes (Treg) may play a pivotal role in the pathogenesis of the disease. In fact, the balance between regulatory and effector functions is important for maintaining efficient immune responses, while avoiding autoimmunity. Indeed, the hyperproliferation of pathogenic effector T cells in psoriasis has been associated with either a reduction or a functional impairment of blood and tissue Treg cells [28, 29].

The therapeutic approach to psoriatic patients is based on two major categories of drugs, namely, the conventional immunosuppressive drugs (i.e., methotrexate cyclosporine) or acitretin and the last generation biological agents. In addition to TNF-*α* antagonists such as infliximab (a chimeric monoclonal antibody composed of a human IgG1 constant region and a murine variable region), etanercept (a soluble TNFR, made of two extracellular domains of the human TNFR2 fused to the Fc fragment of human IgG1), or adalimumab (a human monoclonal antibody), a new drug (ustekinumab), an antibody targeting the common p40 subunit of IL-23 and IL-12, has been introduced in the therapeutic management of psoriasis [30, 31]. The advent of biological drugs has greatly improved the therapeutic management of psoriasis [32]. However, psoriasis has proven to be a difficult therapeutic challenge and treatment failures, even with newer biologic therapies, are not uncommon [33]. Thus, the identification of laboratory parameters for use as surrogate biomarkers for disease assessment and monitoring of therapeutic efficacy, including information about long-term immunological safety, should represent a valuable tool to assist in the clinical and therapeutic management of the disease. To this aim, we have evaluated different immunological parameters in patients affected by moderate to severe psoriasis undergoing systemic treatment with biologic drugs in a controlled clinical study, aimed at assessing the efficacy of different treatment, in order to identify immunologic profiles useful for disease assessment and therapeutic management of patients.

## 2. Materials and Methods 

### 2.1. Study Design

An open prospective observational study (n. RS0209, Ethical Committee Approval n. 64/109), designed to assess the efficacy of therapeutic regimens based on the administration of anti-TNF-*α* drugs (etanercept, adalimumab, and infliximab), was performed in two clinical centers (Tor Vergata University of Rome and the San Gallicano Dermatology Institute) in Rome, Italy, after approval by the institutional ethical committees and in accordance with the Declaration of Helsinki. A further objective of the study, which included patients affected by moderate to severe psoriasis, was to explore different immunological parameters to assess their potential for use in the clinical assessment and therapeutic management of patients.

### 2.2. Study Population

A total of 59 patients affected by moderate to severe active plaque-type psoriasis have been enrolled in the study. The population included 19 female and 40 male patients, aged 46.3 ± 12.3 years. The clinical characteristics are described in Table 1. They did not receive any systemic therapy for at least one month and topical therapy for at least 2 weeks before enrolling in the study. Disease severity was evaluated by the Psoriasis Area and Severity Index (PASI) method [30]. The arthropathy was assessed and periodically monitored through the count of swollen and tender joints [34]. An age and sex matched group composed of 20 healthy subjects undergoing an institutional health surveillance program was used as a reference group for the assessment of the levels of circulating lymphocyte subsets, including regulatory T cells (Treg). All patients as well as reference subjects signed a written informed consent.

### 2.3. Treatment

Patients were assigned to treatment arms with either etanercept or adalimumab or infliximab, according to the criteria indicated by international guidelines [35, 36]. Drugs were administered as follows: infliximab (5 mg/kg administered intravenously at 0, 2, and 6 weeks and thereafter every 8 weeks), etanercept (50 mg subcutaneous twice weekly for 12 weeks and then 25 mg subcutaneous twice weekly), and adalimumab (80 mg subcutaneous at week 0 and then 40 mg every other week). Subcutaneous injections were self-administered by the patients. Clinical monitoring included physical examination and instrumental as well as laboratory diagnostic procedures (X-ray thorax, tuberculosis test, and blood cell test). Disease severity was assessed before therapy (T0) and after 12 (T1) and 24 (T2) weeks. The therapeutic efficacy was defined as the achievement of a reduction of at least 50% (PASI50), 75% (PASI75), or 90% (PASI90), respectively, by PASI evaluation. Eight out of the 59 patients had only arthritis and have not been included in the analysis based on PASI evaluation (Table 2). In arthropathic patients, the improvement of joint involvement was evaluated by clinical examination, by establishing and recording the number of swollen joints.

### 2.4. Laboratory Immune Monitoring

Laboratory monitoring was performed at baseline (T0), 12 weeks (T1), and 24 weeks (T2), respectively.

#### 2.4.1. Soluble Biomarkers

Serum samples were stored frozen at −30°C. Measurements of IL-1*α*, IL-1*β*, IL-2, IL-6, IL-10, IL-12p70, IFN-*γ*, TNF-*α* were performed by a multiplex sandwich ELISA (Human Cytokine Array 1, Aushon Biosystem, USA) and the SearchLight Plus Analysis System and Array Analyst software (Aushon Biosystem), respectively, according to the manufacturer instructions. Concentrations were expressed as pg/mL. Assay sensitivity ranged between 0.2 and 0.4 pg/mL. Detection of IL-17 and IL-22 was performed by ELISA (Platinum ELISA, e-Bioscience, BenderMedSystem GmbH, Vienna, Austria). The sensitivity was 0.5 and 3.4 pg/mL, respectively.

#### 2.4.2. Cellular Biomarkers

Whole blood EDTA (10 mL) was used to assess lymphocyte subsets by flow cytometry (FACSCanto II flow cytometer, BD Biosciences). T, B, NK, and Treg as well as CLA+ T lymphocytes were determined by Multitest TBNK, CD45-PECy7, CD3-PerCyP, CLA-FITC (HECA-452) (Pharmingen-BD Biosciences), and human Treg flow kit (CD4-PECy-5, CD25-PE, FoxP3 Alexa Fluor, BioLegend, San Diego, CA).

#### 2.4.3. Functional Assays

Peripheral blood mononuclear cells (PBMC) were obtained by Ficoll gradient centrifugation (UNI-SEP Tubes, Novamed Ltd, Israel). Proliferation assays toward recall antigens were performed by flow cytometry using 5- and 6-carboxyfluorescein diacetate succinimidyl ester (CellTrace CFSE Cell Proliferation kit, Molecular probes TM, Invitrogen) as described [37]. Briefly, PBMC were washed in PBS w/o Ca++ and Mg++ and stained with CFSE by 10 min incubation at 37°C with 0.4 *μ*M CFSE in PBS supplemented with 0.1% fetal bovine serum (FBS, GIBCO). Staining was stopped by adding 5 volumes of ice-cold complete medium (RPMI with 10% FBS, GIBCO). After washing twice with cold medium, cells were plated in a 96-well microtiter plate at 0.3 × 10^6^/0.2 mL/well. Antigens were tested in triplicate and included* Candida albicans* (5 mg/mL) and CEF peptide pool, comprising Cytomegalovirus, Epstein-Barr, and Flu viruses peptides (2 *μ*g/mL) (Nanogen Advanced Diagnostics). The superantigen Enterotoxin B from* Staphylococcus aureus* (SEB, 0.25 *μ*g/mL, SIGMA-Aldrich) was used as positive control. Negative controls were constituted by triplicate wells with cells plated in complete medium. After 6 days of incubation at 37°C, 5% CO_2_, plates were centrifuged for 4 min at 1200 rpm, washed, and harvested.

Triplicate wells were pooled and stained with a mix of anti-CD4-APC-Cy7, anti-CD8-PE-Cy7, and anti-CD25-APC (BD Biosciences, San Jose, CA, USA). Cells were then washed twice with BD CellWash and analyzed by flow cytometry. The results were analyzed by the ModFit software (Verity Software House, Inc.) and expressed as Proliferation Index (PI), after normalization for the number of acquired events, subtracted from the value of the control sample, and as fold increase (FI), calculated as the ratio of PI in the presence of the specific antigens and the PI values of control samples. Proliferative responses were considered positive when FI was ³2.

### 2.5. Statistical Analysis

Statistical analysis was performed using the Graph Pad Software Prism 4 (GraphPad Software Inc., San Diego, CA). One way ANOVA (and related nonparametric tests) for repeated measures was used to compare baseline measurements with those obtained during treatment. Comparisons between two groups were performed by *t*-test. The Graph Pad Software Prism test for deviations from Gaussian distribution was performed using the Kolmogorov-Smirnov test (normality test).

## 3. Results 

### 3.1. Clinical Efficacy

Table 2 shows the response rate for PASI50, PASI75, and PASI 90 after 12 and 24 weeks of treatment in the all patients group and for each group of treatment. The best clinical efficacy was achieved with infliximab and adalimumab which showed the highest responder rates at 12 and 24 weeks. Etanercept effect was less pronounced at earlier time points and became evident after a longer time of observation.

### 3.2. Soluble Biomarkers

**Figure 1:**
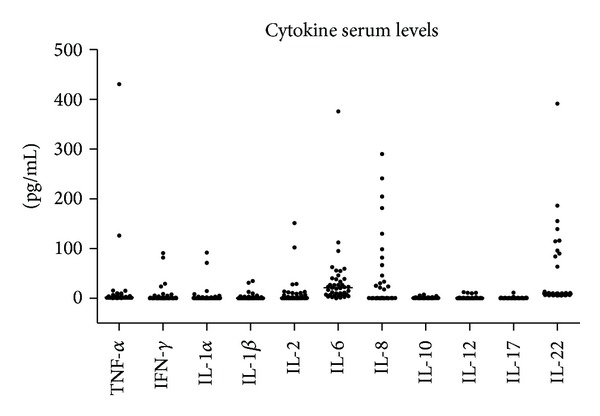
Individual levels (dots) and median values (line) of TNF-*α*, IFN-*γ*, IL-1*α*, IL-1*β*, IL-2, IL-6, IL-8, IL-10, IL-12p70, IL-17, and IL-22 in sera collected at baseline (T0), before therapy initiation.

**Figure 2:**
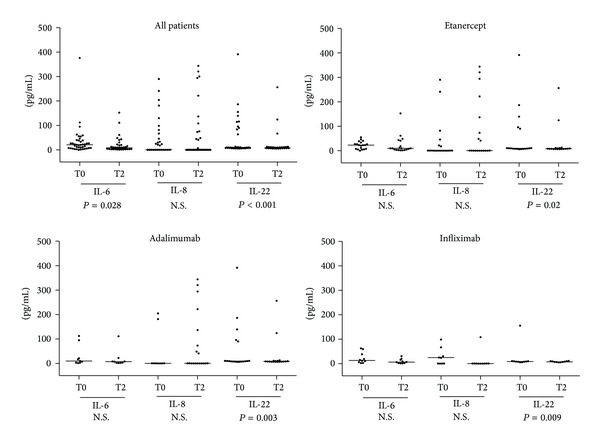
Analysis (nonparametric *t*-test for repeated measures) of the serum levels of IL-6, IL-8, and IL-22, as determined at baseline (T0) and after 24 weeks (T2) of therapy in all patients and in subgroups of subjects stratified according to the administration of the different anti-TNF-*α* drug regimens.

The measurement of TNF-*α*, IFN-*γ*, IL-1*α*, IL-1*β*, IL-2, IL-6, IL-8, IL-10, IL-12p70, IL-17, and IL-22 in sera collected before therapy showed an increase in the levels of IL-6, IL-8, and IL-22, as shown in Figure 1. Consequently, these cytokines were assessed during the therapeutic follow-up. The results, summarized in Figure 2, showed a significant reduction of IL-6 and IL-22 induced by therapy after 24 weeks of treatment in all patient groups, while the levels of IL-8 were not significantly modified. The reduction of IL-22 was found in all treatment groups, while IL-6 reduction was statistically significant only when the analysis was performed considering all patient groups.

### 3.3. Cellular Biomarkers

**Figure 3:**
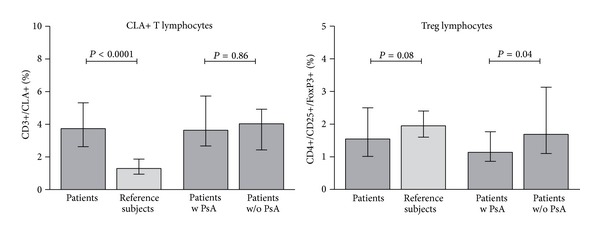
Comparison (unpaired, nonparametric *t*-test) of baseline values of peripheral blood CD3+CLA+ or CD4+/CD25+/FoxP3+ T cells (Treg) in overall patients and a group of healthy subjects or in psoriatic patients with (w) or without (w/o) joint disease. Depicted values are median and interquartile ranges.

**Figure 4:**
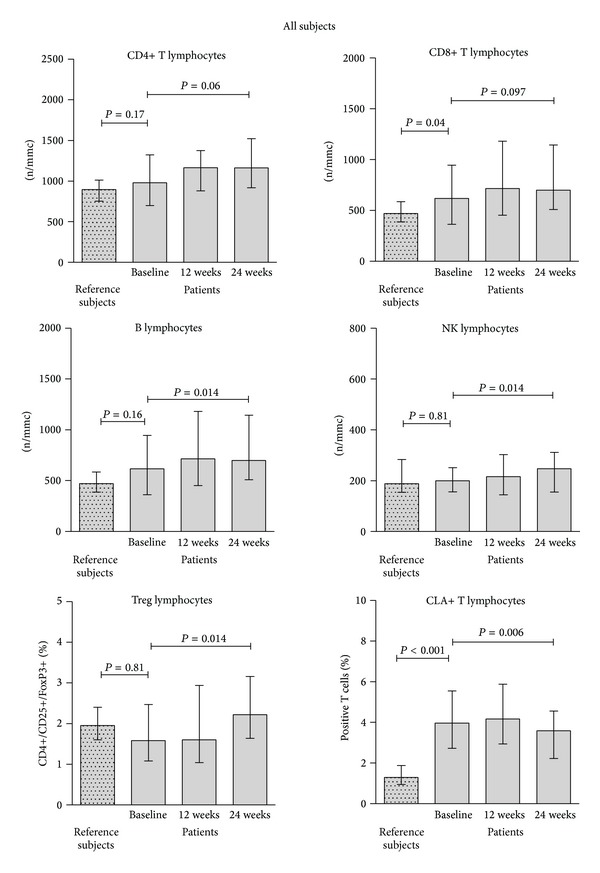
Analysis of lymphocyte subsets (T, B, NK lymphocytes, CD3+CLA+ and Treg cells) in the reference group and in patients before and during anti-TNF-*α* treatment. Baseline measurements in patients and reference subjects were compared by unpaired, nonparametric *t*-test. Measurements before and during therapy were compared by one way ANOVA for repeated measures, nonparametric test. Depicted values are median and interquartile ranges.

**Figure 5:**
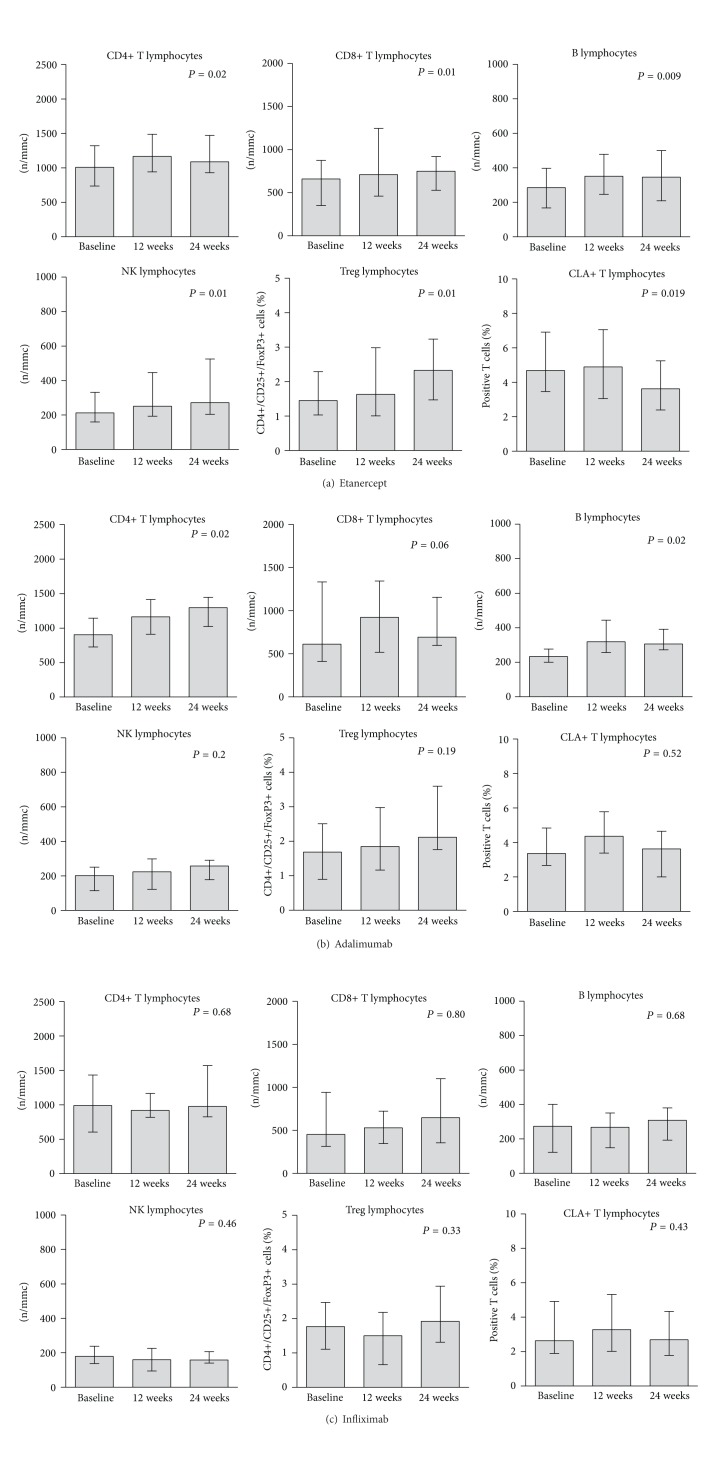
Analysis of lymphocyte subsets (T, B, NK lymphocytes, CD3+CLA+ and Treg cells) in peripheral blood of psoriasis patients before and during therapy with etanercept (a), adalimumab (b), and infliximab (c). Measurements were compared by one way ANOVA for repeated measures, nonparametric test. Depicted values are median and interquartile ranges.

Determination of lymphocyte subsets at baseline showed that patients with psoriasis had higher CLA+ T cells and lower Treg lymphocytes, compared to the reference subjects (Figure 3). The difference was highly significant for CLA+ T cells: median values and ranges were 3.7% (0.9–8) in patients and 1.3% (0.5–2) in the reference subjects, *P* < 0.0001. A borderline significance was found for Treg cells: median values and ranges were 1.55% (0.2–5.4) in patients and 1.95% (1.1–3.2) in the reference group, *P* = 0.08. However, Treg levels were significantly lower in patients with joint involvement 1.14% (0.1–3.7) as compared to patients without arthritis, 1.69% (0.4–5.4), *P* = 0.04, while no difference was observed considering CLA T cells: 1.4% (0.2−3.7) in patients with and 1.7% (0.4–5.4) in patients without joint involvement, *P* = 0.86. The analysis of T cell subsets, B cells, and NK lymphocytes at baseline did not show significant differences as compared to the reference subjects (Figure 4). However, a slight increase of NK and B cells was observed during therapy at 24 weeks, particularly considering the etanercept (Figure 5(a)) and adalimumab (Figure 5(b)) drug regimens, and was not seen under infliximab administration (Figure 5(c)). Interestingly, a significant decrease of CLA+ T cells and a concomitant increase of Treg cells were observed during 24 weeks of therapy (Figure 4), particularly at 24 weeks (Figure 4). These effects were significantly associated with the etanercept treatment (Figure 5(a)) and were less evident under adalimumab (Figure 5(b)) or infliximab (Figure 5(c)) therapeutic regimens.

### 3.4. Response to Recall Antigens

**Figure 6:**
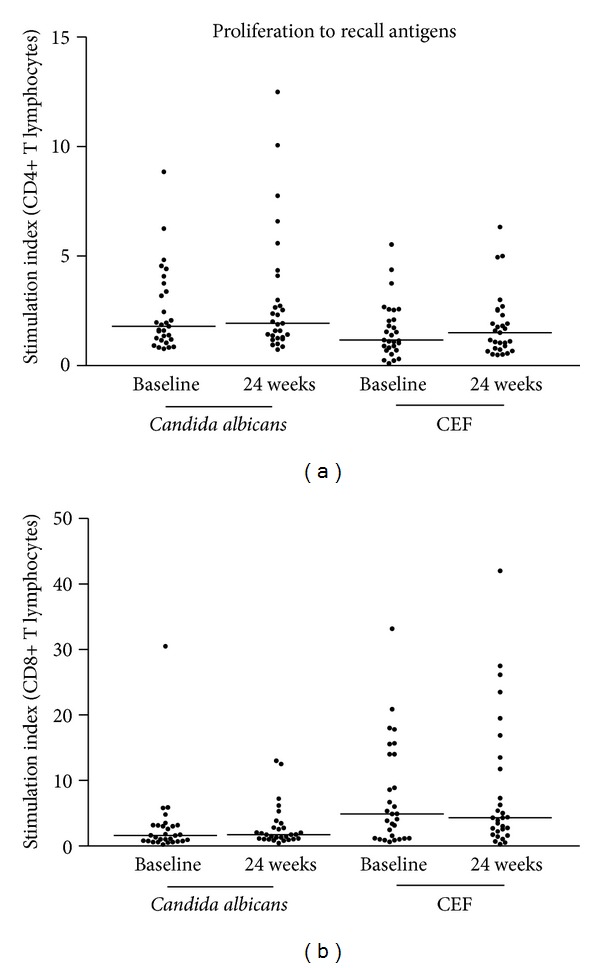
Lymphocyte proliferation toward* Candida albicans* and CEF antigens as performed at baseline (T0) and after 24 weeks of treatment (T2) in a representative group of 30 patients under therapy with etanercept (10), adalimumab (10), or infliximab (10). Stimulation indexes of CD4+ (a) and CD8+ (b) lymphocytes.

To assess the integrity of the adaptive immune responses under drug regimens which target key immune circuitries, proliferation assays toward recall antigens were performed at baseline and after 24 weeks of treatment. The results showed that both CD4 and CD8 responses against* Candida albicans* as well as Cytomegalovirus, Epstein-Bar, and Flu viruses were conserved or even increased under therapy (Figure 6).

## 4. Discussion 

Several studies indicate that biological treatments for psoriasis have a higher clinical efficacy in comparison with conventional drugs [32]. Results gathered in the present study showed that the inhibition of TNF-*α* led to a clinical improvement (PASI50-90) in 87% of patients. Considering each therapeutic regimen, we found that adalimumab and infliximab induced the higher response rate (PASI90 in 90% of patients) already at week 12, while clinical improvement under etanercept regimen was less pronounced at 12 and 24 weeks. However, it induced a more significant modification of T cell subsets, as compared to the other two drugs. This might be explained by the fact that, according to recent studies, etanercept has been shown to induce early biochemical and cellular effects, which precede the clinical improvement [38, 39].

Many studies have pointed out the role of cytokines and angiogenic factors along with the upregulation of the Th1 and Th17 immune response and a downregulation of T regulatory cells in the pathogenesis of the psoriatic lesions [22, 23, 40] suggesting the potential for their use as biomarkers of clinical response to therapy [41]. The analysis of cytokine levels in our patients at baseline showed increased levels of IL-6, IL-8, and IL-22. These cytokines were thus monitored during therapy. Increased IL-8 may represent a relevant sign of disease activity, since it is produced by keratinocytes in response to IL-1 or TNF-*α* [42] and, as a chemokine, has relevant proinflammatory and angiogenic properties [43, 44]. However, serum levels of IL-8 did not decrease in concert with the clinical improvement, in our patients. We rather found a significant reduction of both IL-6 and IL-22 levels during therapy. Our data thus confirm the involvement of Th22 cells in psoriasis, as recently suggested by other authors which also found increased serum levels of IL-22 in the presence of anincrease of IL-6 and in the absence of IL-17 increments [45]. In fact, IL-6 and TNF-*α* promote priming of Th22 [46]. IL-22 is a member of the IL-10 cytokine family, described as having proinflammatory activities on liver, pancreas, intestine, and skin [47]. IL-22 production is upregulated in psoriatic lesion, due to infiltrating T effector cells, which are a major source of IL-22 [27, 28]. Binding of IL-22 to its receptor leads to the activation of the Janus kinase and the signal transducer and activator of transcription STAT3 and of the mitogen-activating peptide kinase pathways [48]. Of note, keratinocytes express high levels of IL-22 receptors and are highly responsive to IL-22 [26]. On the other hand, elevated levels of IL-6 have been reported in psoriasis either in serum or in lesions [7, 40]. IL-6 pleiotropic effects include stimulation of epidermal keratinocyte hyperplasia as well as promotion of the differentiation of IL-17-producing T lymphocytes and inhibition of Treg differentiation [49, 50]. Thus, the significant decrease of IL-6 and IL-22 levels in association with the clinical improvement of patients suggests that an at least partial restoration of an effective immune regulation has been induced by the therapeutic regimens. Indeed, the analyses of regulatory and effector T lymphocytes, that is, T cells expressing CLA as a skin homing receptor, provide some support to this hypothesis. In fact, expression of CLA plays an important role in the homing of memory T cells to the skin, which, in turn, is essential for long-term immune surveillance and the maintenance of barrier integrity [13]. Over 80% of T cells infiltrating the skin express CLA when compared to less than 20% and 5% in the peripheral blood or in tissues other than skin, respectively [16]. CLA+ T cells increase in evolving psoriatic lesions, as compared with stable lesions as well as uninvolved skin. This accumulation of CLA+ cells precedes epidermal hyperproliferation suggesting that these cells may play a key role in the pathogenesis of psoriasis [15] since in psoriasis CLA+T cells include self-antigen-reactive T cells and IL-17 producing cells [51, 52]. In psoriasis, circulating CLA+ T cells correlate with disease severity and a reduction in CLA expressing cells has been reported in association with a clinical improvement after methotrexate treatment [16]. In the present study, we found a significant reduction of circulating CLA+ T cells after 12 and 24 weeks of therapy, particularly under etanercept regimen, which is administrated through intradermal injections. The reduction of CLA+ peripheral T cells may reflect a reduction in keratinocyte activation, since etanercept's earliest target appears to be epidermal keratinocytes [39]. On the other hand, regulatory T cells play a crucial role in the processes of peripheral tolerance and are pivotal for controlling the outgrowth of potentially pathogenic self-reactive T cells [28]. Lower levels of Treg cells in both skin and peripheral blood have been shown in progressive disease while higher levels were found in stable or regressive lesions [53, 54]. In the present study, Treg levels at baseline were lower, though not significantly, than those found in the reference group. However, patients with PsA had the lowest number of Treg cells at baseline, significantly below the levels found in patients without PsA as well as in the reference group. Remarkably, after 24 weeks of therapy, Treg were increased in peripheral blood, in association with the other parameters of clinical efficacy. Most pronounced results were obtained upon etanercept administration. Previous studies have shown that anti-TNF-*α*-based treatment induces an increase of Treg lymphocytes in patients with psoriasis [55]. Indeed, a recent study has shown that TNF-*α* inhibits the suppressive function of Treg cells* in vitro* [17]. Thus, according to this notion, the increments of Treg cells under anti-TNF-*α*-based treatment may represent a key factor in restoring a more appropriate regulation of the adaptive immune response with an effective suppression of CLA+ autoreactive T cells.

The assessment of immunological safety, that is, the preservation of the integrity of immune functions, was performed by assessing peripheral lymphocyte subsets as well as the response to recall antigens. T, B, and NK cell counts at baseline did not significantly differ from those of an age and sex matched reference group. The administration of anti-TNF-*α*-based therapy did not alter either CD4 or CD8 numbers. Conversely, a slight increase in B and NK cells was observed, though with some differences among the different drugs. In particular, etanercept induced mainly increases of NK cells while Humira induced mainly B cell increments. The increment of NK cells, which are key components of the innate immune defense system [55], is intriguing, since NK cells have been shown to exert also regulatory functions on antigen-specific T and B cell responses, although their role in psoriasis has been poorly explored, as yet [56, 57].

Regarding the potential influence of anti-TNF-*α*-based therapy on the setting of adaptive immune functional responses against viral and bacterial antigens, previous reports in different inflammatory diseases, including psoriasis and psoriatic arthritis, rheumatoid arthritis, spondyloarthritis, and Crohn's disease, showed that most common adverse events are represented by the occurrence of infections and by an unexpected high frequency of reactivation of latent tuberculosis infections (LTB), which are often severe and characterized by extrapulmonary and disseminated lesions [58].

Our data, however, suggest that functional immune responses against different recall antigens are remarkably preserved and possibly even increased during a 24-week follow-up of anti-TNF-*α* administration.

## 5. Conclusions

The clinical efficacy of anti-TNF-*α* drugs in patients with psoriasis and/or joint disease was associated with (i) reduction of IL-6 and IL-22 in sera; (ii) increments of regulatory T cells; (iii) reduction of cutaneous homing receptor expressing T cells. This finding indicates that these laboratory parameters may represent useful surrogate biomarkers for the clinical monitoring and therapeutic management of psoriasis.

## Figures and Tables

**Table 1:** Clinical characteristics of patients and treatments.

Therapy (patients)	*T*	PASI 50 patients %	PASI 75 patients %	PASI 90 patients %
All treatments (51)	12 weeks	12	3	31
		*23.5% *	*5.9% *	*60.8% *
	24 weeks	4	6	39
		*0.7% *	*10% *	*76% *

Etanercept (27)	12 weeks	12	2	9
		*44.4% *	*7.4% *	*33.3% *
	24 weeks	4	5	17
		*14.8% *	*18.5% *	*62.1% *

Adalimumab (11)	12 weeks	0	1	10
		*0.0% *	*9.0% *	*90.1% *
	24 weeks	0	1	10
		*0% *	*10% *	*90% *

Infliximab (13)	12 weeks	0	0	12
		*0.0% *	*0.0% *	*92.3% *
	24 weeks	0	0	12
		*0.0% *	*0.0% *	*92.3% *

**Table 2:** Clinical efficacy of TNF-*α* inhibitors on skin involvement.

Therapy	Patients	Skin involvement	Skin and joint involvement	Joint involvement
All patients	59	32	19	8
		∗PASI = 12 (8.5–39.6)	PASI = 10.35 (1.8–34.2)	

Etanercept	29	17	10	2
		PASI = 12 (8.5–26)	PASI = 12 (1.8–15.3)	

Adalimumab	15	7	4	4
		PASI = 12.2 (10.3–33)	PASI = 15 (13–15)	

Infliximab	15	8	5	2
		PASI = 12 (10–39.6)	PASI = 5.8 (4–34.2)	

*Median and range.
